# Commentary: Trim the fat

**DOI:** 10.1016/j.xjtc.2021.11.008

**Published:** 2021-11-11

**Authors:** Thomas E. MacGillivray, Michael J. Reardon

**Affiliations:** Department of Cardiovascular Surgery, Houston Methodist Hospital, Houston, Tex

**Figure:**
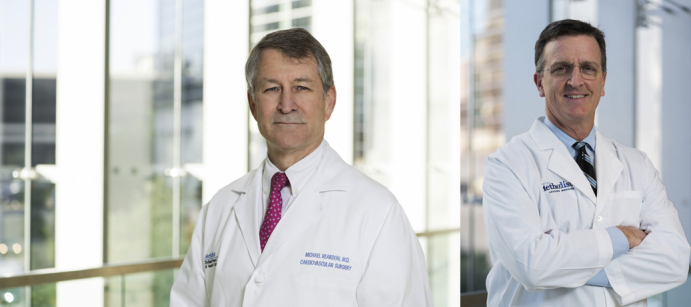
Michael J. Reardon, MD, and Thomas E. MacGillivray, MD

**Central Message**

Complex cardiac tumors present a challenge in evaluation and management. A multidisciplinary approach and proper use of imaging is imperative. 3-dimensional model use can be helpful in these cases.

See Article page 37.

Primary cardiac tumors are rare, and most surgeons gain limited experience in their management. Most benign tumors such as myxoma and fibroelastoma can be surgically resected with excellent outcomes and minimal to no mortality.^1^ More complex benign tumors such as paraganglioma generally require more planning and experience for successful surgical resection.^2^ Malignant primary cardiac tumors are substantially more complex to evaluate and treat. The most common malignant primary cardiac tumor is sarcoma, and surgical resection has been associated with increased survival in this deadly disease.^3^ The second most common malignant primary cardiac tumor is lymphoma, and surgical resection has no survival advantage and should be avoided.^4^ Primary sarcomas without metastatic disease are best treated with complete surgical resection when anatomically and physiologically possible.^5^ Even with excellent imaging, the anatomy may be challenging, and additional measures to fully understand the tumor anatomy and involved cardiac structures may be beneficial in complex tumors.

In this issue of the *Journal*, Kim and colleagues^6^ present an unusual large cardiac tumor penetrating the right ventricular wall and their approach to evaluation and treatment. Their patient was a 33-year-old woman who had a large cardiac tumor found incidentally on echocardiogram. There was no ventricular or valvular function impairment by the tumor. Computed tomography showed an epicardial-based tumor penetrating into the right ventricular wall on the inferior surface of the heart. The working diagnosis was liposarcoma, and surgical resection was planned aided by the 3-dimensional printing of a model to allow advanced surgical consideration. The surgical procedure is nicely described in the text and well-illustrated in the companion video. The surgical pathology as well as the image of the tumor at surgery are both consistent with a cardiac lipoma, and the patient did well and should be cured. The authors are to be congratulated for this excellent outcome.

Despite this positive outcome for the patient, there are several points worth discussing about the case. Primary tumors of the heart believed to be malignant would be best managed in a center with a dedicated cardiac tumor team.^7^ We have employed a cardiac tumor team from MD Anderson Cancer Center and the Houston Methodist Hospital that meets monthly with a focus on complex and malignant primary cardiac tumors. This has led to an experience of surgical resection in 122 primary cardiac sarcomas.^8^ An advantage of a dedicated team is a more in-depth understanding of the needed imaging and its interpretation. One of the more common scenarios we are presented with is the incomplete resection of a left atrial sarcoma that was believed to be a large benign myxoma on image interpretation. Differentiating benign from malignant primary tumors requires multimodality imaging and in-depth knowledge of proper interpretation.^9^^,^^10^ Suspecting a primary cardiac tumor, especially when on the left side of the heart, to be malignant helps the surgeon plan for the advanced surgical techniques that may be necessary for complete surgical removal.^11^ In the case presented, a malignant liposarcoma was suspected and a benign lipoma was found. Although the patient did well, cardiac lipomas do not always need to be resected, and we have followed several without tumor growth or consequence. Cardiac magnetic resonance imaging is very helpful in this regard. Lipomas generally have homogenous high signal on T1 with complete suppression with fat saturation and no low signal on T2 with fat saturation. Liposarcomas tend to be larger, more irregular, and have a heterogenous signal on T2 with fat saturation. This of course is not always definitive, but it would be of interest to review the cardiac magnetic resonance imaging in this case. Finally, we fully agree with the authors that 3-dimensional printing can be very useful in evaluating the surgical approach to complex tumors and have also previously used this to our benefit.^12^

We again congratulate the authors on an outstanding case and excellent result. Every addition to our knowledge base for complex primary cardiac tumors helps advance our field and benefit our patients.
